# Visualise the tastes from the label: A study on the taste-colour crossmodal association of crisp and dry

**DOI:** 10.3389/fpsyg.2022.980049

**Published:** 2022-09-28

**Authors:** Mengmeng Wang, Dongning Li

**Affiliations:** School of Design, Jiangnan University, Wuxi, China

**Keywords:** colour design, crossmodal association, beer, taste, label design

## Abstract

Colour is an important guideline for selection and consumption. It also draws attention to the designers, as some modern design styles require them to illustrate the taste of the product with a limited number of colours. In this case, a precise description of the taste-colour association is required. The present study explored the colour-taste crossmodal association of two tastes, crisp and dry, which are normally found in beers and are the preferred flavours of Chinese consumers. Experiments were carried out to determine the characteristics of the colours associated with the two tastes. And the strength of the tastes perceived from the colours with different hue angles was investigated. The results of this study reveal that the hue and chroma can both affect the perception of these tastes. Both tastes can be perceived from the same colour, but the strength of the taste can be varied from different hues.

## Introduction

Colour is vital information for humans to evaluate foods and beverages before consuming them. From the sweetness of the yoghurt to the flavour of the bottled drinks, the colours of the package deliver essential information of the foods/liquids inside the containers and can be used to influence the consumption behaviour of customers. For food/beverage packages, a previous study claims this taste-colour correspondence, as a crossmodal perception, relates to the flavour, sound, or aroma, where the colour reflects the multisensory character of the food and beverages (Spence, 2013; Reinoso Carvalho et al., 2016; Wang et al., 2019). On the other hand, colours can also affect the expectation and orosensory perception of the beverages/foods (Piqueras-Fiszman and Spence, 2015; Velasco et al., 2016; Spence, 2019a). This influences both the single sense and the general perception. Research also reported that the taste-colour correspondence is associated with the colour meanings which relate to different consumer expectations (Wei et al., 2014). This suggests that different types of foods/beverages can have the same taste, but are associated with different colours, and vice versa. Our trust in this colour to taste perception is found to be universal, which can relate to the generations of training in evaluating fruit ripeness (Maga, 1974; Spence et al., 2015; Foroni et al., 2016). But it is also found to be related to the type of the foods, the geographic locations, and cultural background (Wan et al., 2014; Spence, 2021). Overall, colour is an effective element which can be used to guide the consumer for food/beverage consumption. But the association between colours and tastes is complex. Many traits can affect this association and lead to different perception results.

Even though the relationship between colour and taste is not clarified, numerous research findings show encouraging suggestions to the graphic designers to use the extrinsic factors of the product to convey the sensory, such as the taste, smell, or sound, through the different colour schemes. Early research shows that 15% more yellowness in the colour of the can is perceived to have a more intense lemon flavour (Cheskin, 1957; Spence, 2016). Two opposed colours, red and blue, can result in different sweetness and intensity of the yoghurt flavour (Tijssen et al., 2017). A similar effect is found in the colour of the food packages (Huang and Lu, 2015). This colour to taste association influences is not only found in the pre-tasting test but also impacts the post-tasting test results (Carvalho and Spence, 2019). The colour of the package was also found to be effective in building a connexion with trending food concepts, such as health, and organicness (Huang and Lu, 2015; Bou-Mitri et al., 2020; Plasek et al., 2020), which is found to be more effective than the text information on the package for the young consumers (Vila-López and Küster-Boluda, 2018).

As the popularity of the minimalism package design keeps rising, colour becomes a particularly concerning element in the package design. The minimalism package design aims to keep consumers focused on the major property of the product with a minimum number of colours. Therefore, it is crucial for the designer to understand what the colour means to the consumers before using it. Investigations were carried out to determine the properties of the colours (such as hue, brightness, and saturation) and their meanings to the consumers (Spence, 2015). Some studies were carried out to determine the basic tastes and their associated colours. The major focus of these studies is the relationship between taste and hue. For example, green is found to be related to sour. Pink, orange and red relate to the sweet (Tomasik-Krótki and Strojny, 2008; Wan et al., 2014; see Spence et al., 2015, for a review; Spence, 2019b). The influence of brightness and saturation on perceived tastes was also investigated. The sausage package with a lower brightness is percieved as fattier. The dairy product is perceived as creamier with higher brightness packaging (Tijssen et al., 2017). In summary, srtudies outline a critical role for the colour package design. The findings demonstrate that colour and the consumers' expectations are closely related. Also, this colour-taste association can be affected by the product category, which can result in the same colour relating to different tastes.

Craft beer is a beverage containing a large variety of types and tastes which is starting to get popular in China. Most craft beer types have more complex layers of tastes than industrial beers, which can lead to a strong positive/negative reaction for new consumers. To recommend craft beers that can match consumer preferences, retailers use colours to label the craft beers. For example, the LCBO provides a beer guide, which labels the craft beers according to 3 different bodies and 6 different flavours/aromas. The consumer can use this guide to find the beer that matches their preference (Brown, 2016). However, the taste of craft beer is complex, and hard to be identified through 3 bodies and 6 flavours/ aromas. Also, different brands use different brewing yeast and recipes to manufacture, which can result in the beers being filed in the same category but tasting differently. Apart from the works carried out by the retailers, the taste-oriented colour scheme design was investigated by the researcher and beer companies to find a possible solution for describing beers. An early design case demonstrates that the colour scheme of the beer label can be a major graphic factor causing negative associations (Favre and November, 1979). Further studies were carried out to determine the relationships between the colour of the package and the preference for the beer. The colour of the labels on the bottle of beer is found to have a significant impact on the perceived flavours, which can influence the consumers' purchase intention (Barnett and Spence, 2016). The colour of the cans shows a significant influence on the pre-tasting test. And the agreement between the expectation from the colour of the package and the actual taste is found to have a great influence on the consumers' preference and willingness to purchase (Liu and Oh, 2021). Overall, the studies reviewed here show that colour is a possible way to deliver flavour through the beer package. They found the package colour can affect the perceived taste of beer, which leads to the difference in preferences.

In our previous unpublished survey, we found two tastes of the beer, crisp and dry, are considered as important traits in beer consumption for Chinese young people. These two tastes both can be found in craft beers and are hard to distinguish through the Chinese text message. Therefore, in this study, we explore the colour-taste crossmodal association of these two beer tastes, crisp and dry, and investigated whether the degree of these two tastes can be visualised through the colour-taste association. Two experiments were carried out in this study. Experiment 1 was designed to determine the perceived colours of crisp and dry. Their characters and their relationships were investigated. Experiment 2 was designed to test the colour-taste association in beer label design. The tastes associated with colours were evaluated by applying them to the beer label. As these tastes are hard to distinguish, and the related colour might appear to be similar, this experiment can validate the limits of using the colour-taste experimental results in beer label design.

## Experiment 1

### Material and experimental procedures

Two craft beer samples were provided to the participant, which were used to assist the participant to refresh the taste of crisp and dry. These samples were evaluated and selected by a professional beer sommelier, where sample A has a strong crisp taste and sample B has a strong dry taste. Both samples were distributed in a covered sample cup, and 15 ml of each sample was provided. A glass of water was provided to help the participant neutralise the taste of the beer. The participant was asked to taste the beer samples in random order. After tasting a sample, the participant was asked to select a colour from the display to represent the taste of “crisp” and “dry”.

**Figure 1:**
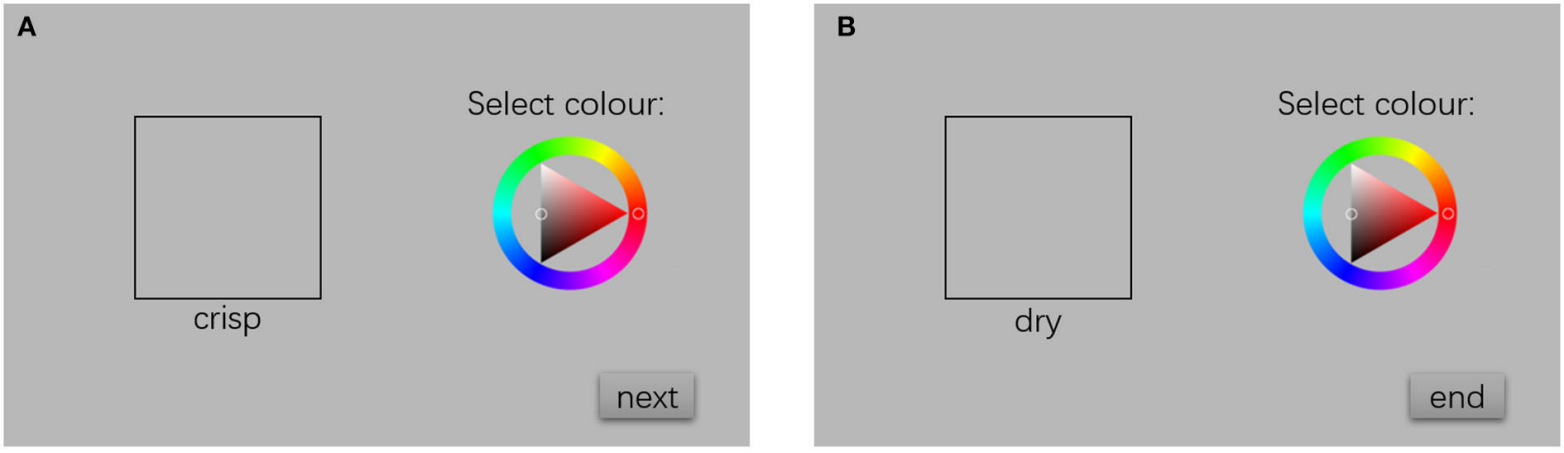
The colour selection interface for **(A)** crisp and **(B)** dry.

Sixty-three participants (19 males, 44 females) aged 24.61, SD = 0.98, voluntarily participated in this study. These participants self-declared that they have experience drinking beers and have no history of allergy. The colour vision of the participant was tested by using an Ishihara colour test plate, and they all passed the test. The experiment was carried out in a standard visual experimental environment which is painted in grey. A characterised display was used in this experiment. Here, Piecewise Linear Chromaticity Constancy (PLCC) model was used to build the colour profile. The experimental interface is shown in Figure 1. A colour wheel, which was built based on the HSB system, was used for colour selection. The selected colour was demonstrated on the left side of the screen, and the initial colour was set the same as the background, which is 50% grey, as shown in Figure 1. The RGB values of these colours were stored, and the characteristic information of this display was used to transform the colour into the CIELAB values (D65, 2°).

### Results and analysis

The experimental results were converted into CIELAB values (D65, 2° standard observers), and the distributions of these results in an a*b* plane are used to analyse the characteristic of the colours. The fitted ellipses, which are marked in orange, are generated based on the experimental results. These ellipses are used to illustrate the distribution of the results. The fit ellipse function which was contributed by Ohad Gal (2022), was used to generate the ellipses. This function uses the Least-Squares criterion for estimation of the best fit ellipse and extracts the parameters from the conic representation of the ellipse. These extracted parameters were used to illustrate the ellipses in the figures.

**Figure 2:**
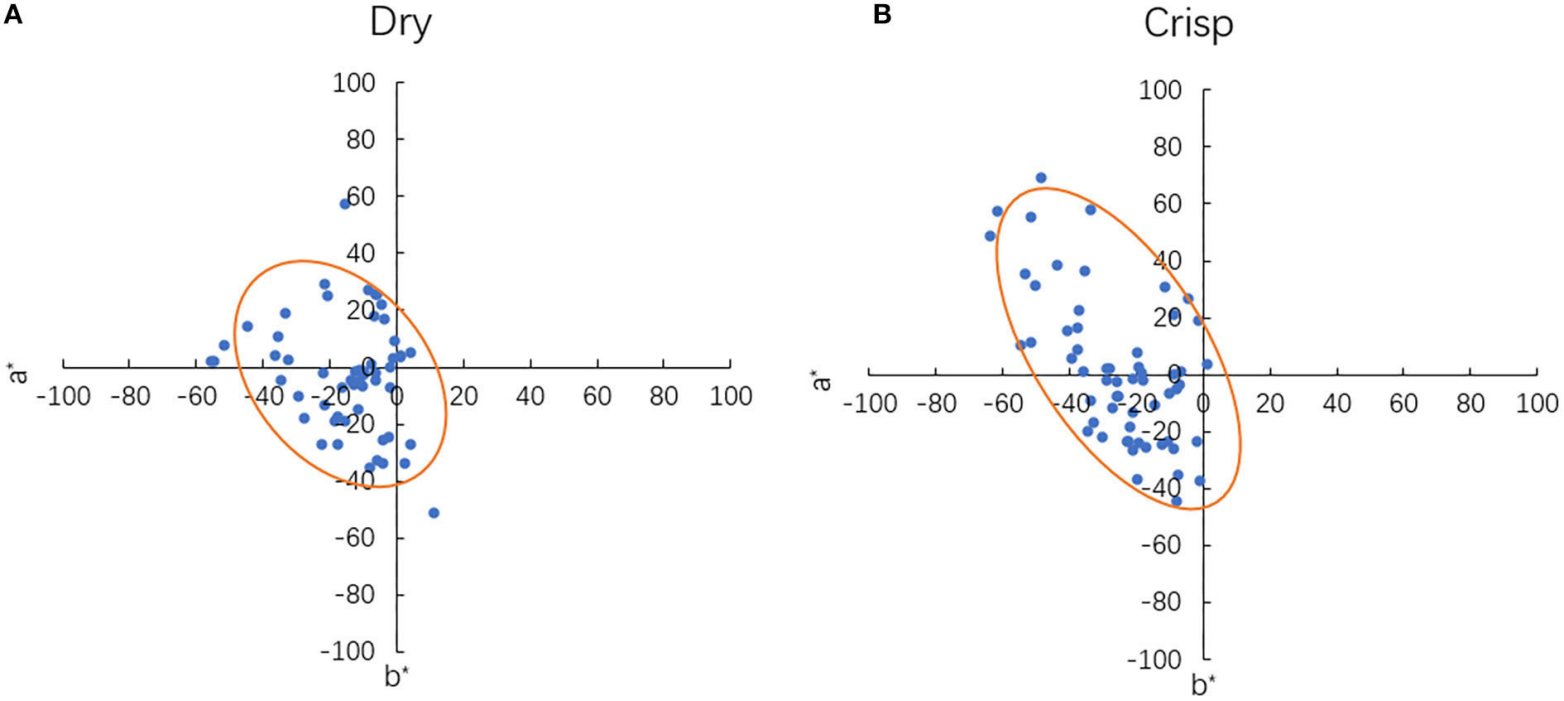
The colour distribution of selected colour in CIE a*b* plane: **(A)** crisp; **(B)** dry.

The crisp and dry taste associated colours (named CTC and DTC, respectively) were distributed in quadrants II and III mostly, as shown in Figure 2. The average hue angles of CTC and DTC are 185.11° (SD = 46.05) and 181.19°(SD = 58.59), respectively. The DTC distribute closer to the origin than that of the CTC. The average distance of the colour data to the origin, the chroma value, is 24.31 (SD = 14.63) and 34.97 (SD = 18.51), for CTC and DTC, respectively. The average of the CTC and DTC appeared to be similar in hue angle value, but the DTC have larger hue angle ranges than that of the CTC. The difference in their average chroma values is about 10, and the DTC has larger chroma ranges than the CTC.

The difference between the colour centre of DTC and CTC (DTC-CTC) in terms of lightness (DL), chroma (DC), hue angle (Dh), and colour difference (DE) are listed in Table 1. The colour differences between two colour centres are calculated by using the CIE DE2000 formula. Their DE indicates that the colour difference between dry and crisp centres are visually distinguishable (DE more than 2). The value of DL is positive, which shows that the colours for dry have a higher average lightness value than the crisp. The negative DC and Dh results indicate that the crisp is blueish and more saturated. These imply that the average colour of dry is brighter and less colourful than crisp. It is also more yellowish-green than that of crisp.

**Table 1:** The difference between the crisp and dry colour centres.

**Dry-crisp**	**DL**	**DC**	**Dh**	**DE**
mean	3.13	−6.69	−3.93	9.05
medium	4.74	−5.37	4.12	11.82

**Figure 3:**
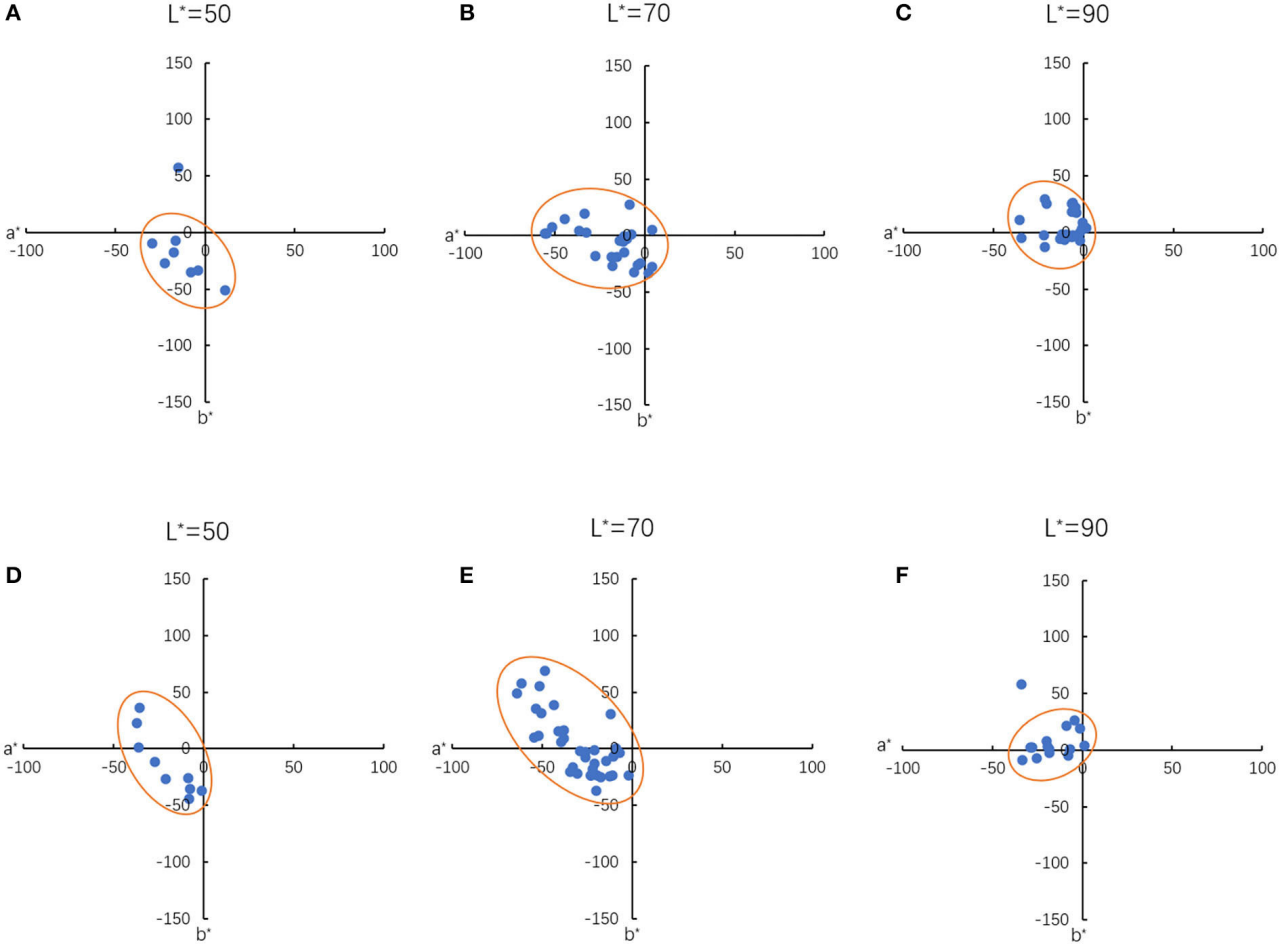
Distribution of the experimental results in different lightness level. For dry at **(A)** L* = 50, **(B)** L* = 70, **(C)** L* = 90; for crisp **(D)** L* = 50, **(E)** L* = 70, **(F)** L* = 90.

The colour distribution of the experimental results at different lightness levels is shown in Figure 3. Here, the data with lightness from 50 to 70, 70 to 90, and above 90 were projected on the a^*^b^*^ planes, with L^*^ = 50, L^*^ = 70 and L^*^ = 90, respectively. The lightness range of the CTC and DTC are similar, which are 81.02 (SD = 11.97) and 84.15 (SD = 11.67), respectively. The data sizes of DTC, which distribute at L^*^ = 70 and L^*^ = 90, are 42.11 and 43.86%, respectively. Most of the CTC distribute at L^*^ = 70, indicating data size is 57.63%. These show that CTC and DTC have different distributions in terms of lightness, even though they have similar lightness ranges. Most of the DTC and CTC lightness is above 70. The majority of the CTC is at a lightness range from 70 to 90. Both the DTC and CTC distribute close to the origin at L^*^ = 90, which can relate to the fusiform shape of the CIELAB gamut. For L^*^ = 50, the mean hue angle for DTC and CTC are 220.67° (SD = 55.12), and 214.94° (SD = 50.91), respectively. For L^*^ = 70, the mean hue angle for DTC and CTC are 199.06° (SD = 53.93), and 189.06° (SD = 39.94), respectively. These results show that the mean hue angle of DTC and CTC at L^*^ = 50 and L^*^ = 70 are similar. But the distributions in the a^*^b^*^ plane show different trends. This can be observed through the fitted ellipse model. The ellipses also suggest that the hue angle ranges of DTC and CTC have an overlap section. According to the ellipse models, the overlap section is from 127.14° to 189.33°. For the chroma, The CTC, which distributes at the quadrant II of a^*^b^*^ plane, has a higher chroma value than that distributed at quadrant III. The chroma value of the DTC is less variable than the CTC in both quadrants II and III.

### Summary

The characteristics of the colour associated with crisp and dry beer taste were investigated in this section. These characteristics were analysed through the distribution of the experimental results in CIELAB space (D65, 2°). And their differences in chroma and hue angle can be observed. The DTC is less saturated and mostly distributed in the ranges of green to blue. The hue range of the CTC is from yellow-green to green-blue. The chroma of the DTC is less variable than the CTC at the QII of the a^*^b^*^ plane. Overall, the trends are found from the distribution of DTC and CTC in terms of chroma and hue, which could be used to visualise the degree of crisp and dry taste of the beer. Experiment 2 is to explore the possibility of using colour to “illustrate” the crisp and dry taste of beer.

## Experiment 2

### Experimental methodology

Fifty-nine participants (16 males, 43 females) aged 25.13, SD = 0.97, voluntarily participated in experiment 2. The participants self-declared that they had experiences drinking beers. The colour vision of the participant was tested by using an Ishihara colour test plate, and they all passed the test. The experiment was carried out in a standard visual experimental environment as experiment 1. The same calibrated display, which was used in experiment 1, was used in this experiment.

**Figure 4:**
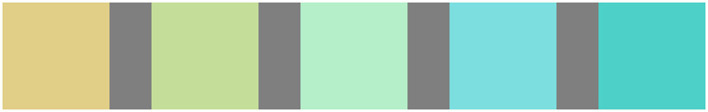
The selected five colours.

Based on the results analysis of experiment 1, the hue angle and perceived crisp and dry taste of the beer were investigated in experiment 2. Five colours were selected from the merged hue angle ranges of crisp and dry, which are from 93.74 to 221.19° with intervals of about 30°, as shown in Figure 4. The lightness and chroma of these colours are the average lightness and chroma values of the experimental results from experiment 1. Then, these five colours were used to design the label of the beer bottle. Here, a national popular beer brand glass bottle package was used as a stimuli model. Six stimuli, including one repeat, were used in this experiment. The stimuli were named S1 to S6 (S6 is the repeated stimulus), which were in the same order as the selected colours in Figure 4.

In experiment 2, the forced-choice categorical judgement method was used to investigate the relationship between the perceived crisp and dry and the label colours. During the experiment, the participant was first required to choose whether crisp/dry can be perceived from the beer package or not. Then the participant scored the degree of crisp or dry according to a 5-point scale, where 1 represents the least and 5 presents the strongest. If the participant selected that the crisp/ dry can be perceived from the stimulus, the score was stored as a positive number. Otherwise, it was stored as a negative number.

### Results and analysis

#### The observer variation

The repeatability of observer judgement was examined through the repeat stimuli. And it was calculated by using the standardised residual sum of squares (STRESS). The observer judgement repeatability for crisp and dry are 27.45 and 34.11 in terms of STRESS value, which is below the STRESS value of normal intra-observer variation at the colour related judgement (Melgosa et al., 2011). This validates the experiment results and also implies that the observer is less variated at judging the crisp from the beer package than the dry.

#### z-score and colour trend

**Figure 5:**
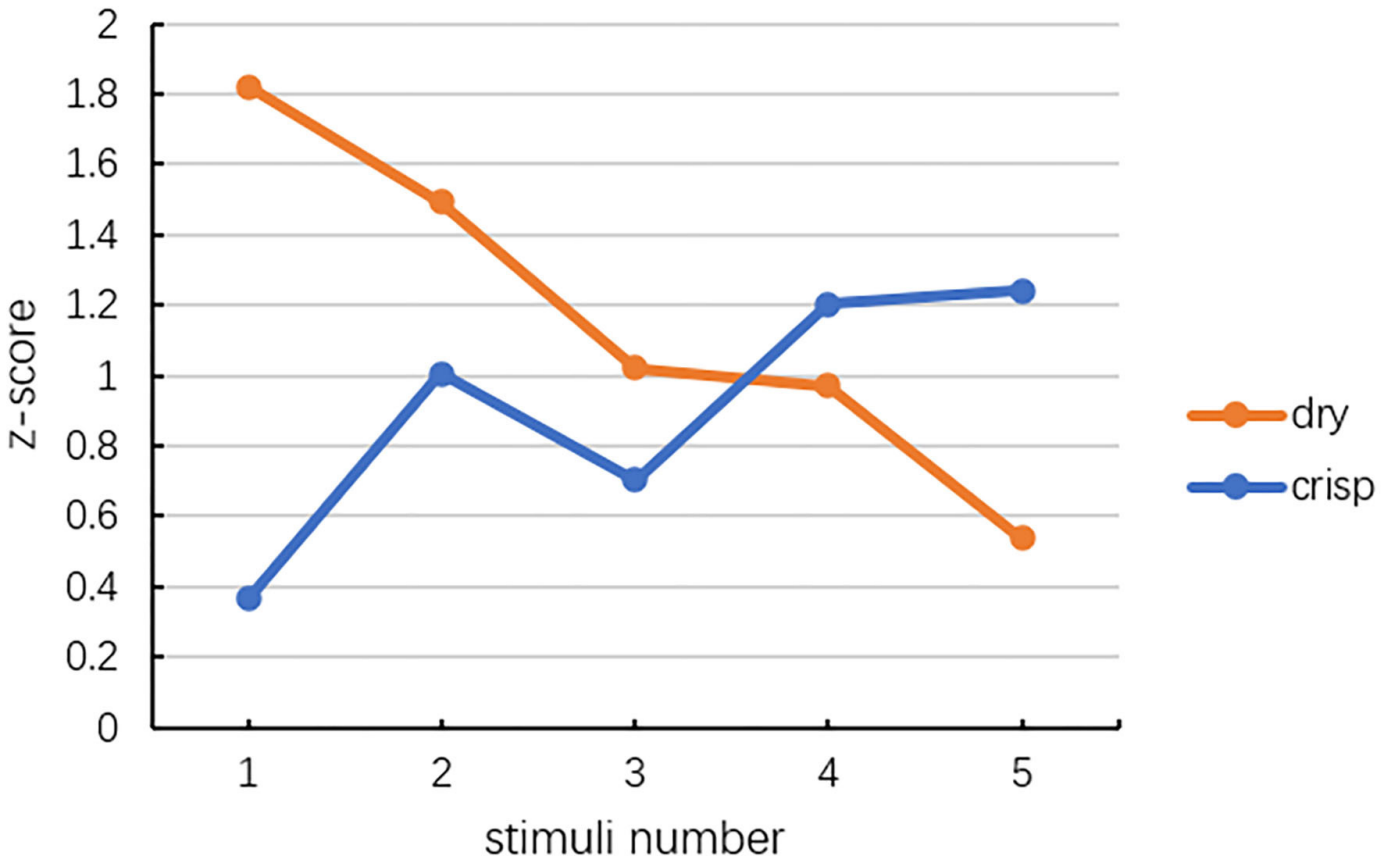
The *z-*scores of five stimuli.

The experimental results are converted into *z-*scores first. The *z-*scores of each stimulus at judging crisp and dry are shown in Figure 5. The *z-*scores of all five stimuli are above 0, which indicates that these five stimuli allow the participants to perceive crisp or dry. From S1 to S5, the z scores of crisp increase and the z scores of dry decrease, as shown in Figure 5. These trends show an agreement with the results of experiment 1, where the crisp taste of the beer can be represented by the colour with a relatively larger hue angle within the range, and the dry taste can be represented by the colour with a smaller hue angle within the range. The z scores of S3 for crisp and dry show a limited agreement to the trend, as the hue angle of the S3 is at the intersection section of the hue angle ranges for crisp and dry.

These results suggest that the crispness and dryness of the beer can be achieved from the colours, and the degree of the crispness and dryness can be visualised by using colours. Also, they show that taste-colour synaesthesia is a possible way to implement in the package colour scheme design to demonstrate the taste of beer.

#### ANOVA analysis

Welch's ANOVA and Games-Howell *post hoc* pairwise comparison was used in this study. The analysations were conducted on the dependent variables “dry” and “crisp”. The hue angle of the label colours was the factor between the stimuli. All significant values were reported at *p* ≤ 0.05.

**Figure 6:**
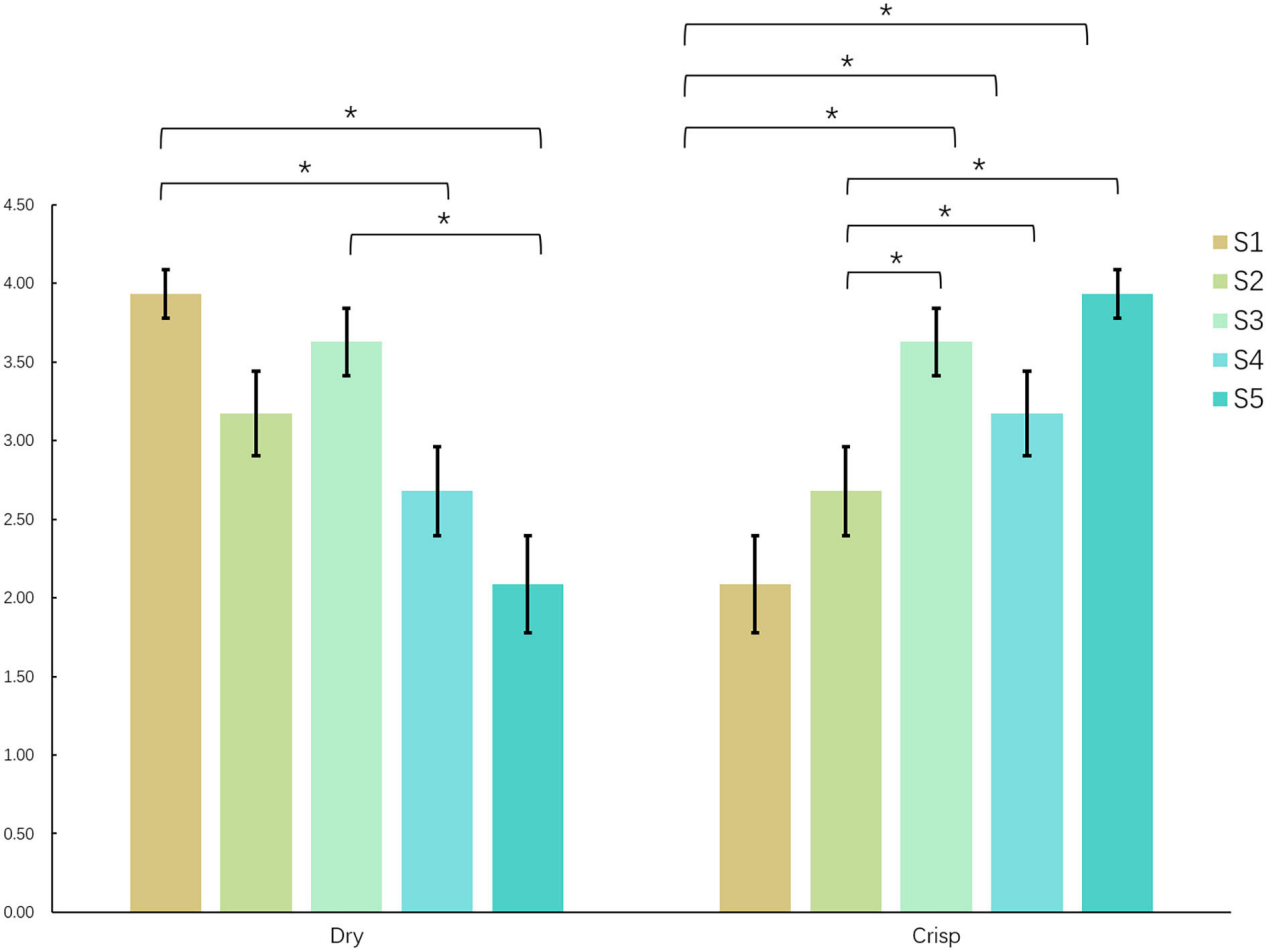
Mean rating (± SE) of five stimuli at dry and crisp. Asterisks indicate statistical significance at *p* < 0.05 (*) (Games-Howell corrected).

The Welch's ANOVA results reveal that the hue angle has a significant effect on the perceived dry taste, F_Welch_(4, 142.208)_= 9.367, *p* < 0.05, η^2^= 0.106, and the perceived crisp taste, F_Welch_(4, 139.932)_= 16.870, *p* < 0.05, η^2^= 0.206. Games-Howell *post hoc* test results reveal that the S1 (2.08 ± 0.31) is perceived as less crisp than S5 (3.93 ± 0.16), S3 (2.63 ± 0.21) and S4 (3.17 ± 0.27). The S2 (2.68 ± 0.28) is less crisp than S4, S3, and S5. The S5 (2.08 ± 0.31) is perceived as less dry than S1 (3.93 ± 0.16) and S3 (3.63 ± 0.21). S1 is perceived as drier than the S4 (2.68 ± 0.28) (see Table 2 and Figure 6). These descriptions also illustrated in Figure 6 which show a trend of decrease in average dry perception and increase in average crisp perception. But the perception of the dry taste is not significant. The increase of the crisp perception while the hue angle changes is significant.

**Table 2:** Compilation of crisp and dry ratings (mean ± SE).

	**Crisp**		**Dry**	
**Stimulus**	**Mean**	**SE**	**Mean**	**SE**
S1	2.08	0.31	3.93	0.16
S2	2.68	0.28	3.17	0.27
S3	2.63	0.21	3.63	0.21
S4	3.17	0.27	2.68	0.28
S5	3.93	0.16	2.08	0.31

### Summary

The strength of the taste perceived from the colours, which change in hue, was investigated in experiment 2. Five colours, which were selected based on the results of experiment 1, were used to design beer labels for this experiment. The experimental results show that these five hues have an influence on the crisp and dry taste. The colour with a larger hue angle is rated as drier or less crisp, and vice versa. Except for the colour with the hue angle at the overlap section of the DTC and CTC, S3. This agrees with the results from experiment 1.

## Discussion and conclusion

Colour has been found to have a close association with different sensory modalities beyond vision (Spence, 2020; Spence and Di Stefano, 2022a), and colour-taste association is one of those occurrences of crossmodal associations. The crossmodal association of colour and taste is encouraged to be used in food and beverage package design by previous research. The colour was found to be valid information in providing a guideline for the consumer to perceive the taste through vision. Previous studies find that the types or the strength of the tastes can be perceived through the colour of the package. But the same colour can be perceived as different tastes when it is applied to the package design of different food/beverage categories. In some cases, the flavours/tastes of the same type of food/beverage can be “sense transferred” into colours which are analogous, or simply monochromatic. These colours are similar and easily misused. To effectively use package colours to guide consumers, further research on the characteristics of the colour and the related flavours/tastes needs to be carried out. The present study was designed to explore the character of the colours between two tastes of the beer, crisp and dry, which can be found in most craft beers. Also, these two tastes are found to be easily confused by Chinese customers, as their Chinese characters and pronunciations are similar (crisp in Chinese as 清冽, dry in Chinese as 清爽). Two experiments are included in this study. Experiment 1 aims to determine the characteristic of the colours that associate with a dry and crisp taste. Beer samples, which were selected by a professional beer sommelier, were used to guide the participants to select the colour to represent their tastes. Based on the characteristics of the DTC and CTC, five colours were selected to generate stimuli for experiment 2. Experiment 2 aims to explore the tastes and their strength perceived from the stimuli.

The results show that two tastes of beer are associated with different colours (average colour difference above 2). These colours have similar lightness ranges but are different in the ranges of hue and chroma. Our results agree with the previous research that hue is the character that can be used to distinguish different tastes (see Spence et al., 2015, for a review). However, the hue angles of the two tastes are adjacent and overlapped. This implies that hue might not be the best way to visually distinguish some of the tastes. Inspired by a recent review in crossmodal harmony from Spence and Di Stefano (2022b), the hue angle range of perceived colour for the crisp and dry might not only reflect the colour of the related subjects, but the colour to promote harmony with the tastes. Also, crisp and dry are not two isolated flavours in craft beer. The balance between them might be a factor to be considered during the colour selection of experiment 1. The DTC and CTC, which are distributed at QII of the a^*^b^*^ plane, are different in chroma. This show that the chroma is not only related to the strength of the tastes (Tijssen et al., 2017), but also can be a character to distinguish different tastes. It is important to mention that the sample selected to guide the participants to select the colour for the taste contains more than crisp and dry. These samples contain similar strengths but different types of malt aroma, also the sample with a stronger dry taste contains a hint of citrus. The participants were guided to experience the crisp and dry, but these unavoidable scents and tastes might influence the experimental results. In these two experiments, the crisp and dry were translated into Chinese and these translated words were consistently used in both experiments. Different translations of these two tastes might affect the experimental results, which plan to be determined in a future study.

For the hue angles, which were further investigated in experiment 2, the crisp and dry tastes can be perceived from both DTC and CTC hue angle ranges. But the colour, with the hue angle within the DTC ranges, was rated as strong in dry taste. So, the colour with the hue angle within the CTC ranges. This suggests that the hue angle can be used to represent different tastes but might not be a reliable factor to be used to differentiate the tastes. The hue angle ranges of these two tastes have an overlapping section. In this study, we only selected one colour from this section, and this colour was perceived to have a stronger dry taste than a crisp taste. We suggest there may be a balance point of hue to represent equal strength of the crisp and dry in the overlapping section. However, as a small number of hue angles were investigated in this study, the current results couldn't support determining this balance point. We expect to investigate the existence of this balance point in our future studies.

The results reported here provide further evidence that the colour is valid information for the consumer to understand and visualise their expectation for the beverages. It is intriguing that the perception of colour to taste is more sensitive than was expected. The colours that are used to guide the consumer need to be carefully examined before applying. As this crossmodal association can be affected by the other external traits (background, environment, cross-cultural, etc.), it will be interesting to explore the relationship between the colour-taste and other traits in the future study.

## Data availability statement

The raw data supporting the conclusions of this article will be made available by the authors, without undue reservation.

## Ethics statement

The studies involving human participants were reviewed and approved by Jiangnan University Ethics Committee. The patients/participants provided their written informed consent to participate in this study.

## Author contributions

MW and DL designed the study. MW collected and analysis the data. All authors contributed to the article and approved the submitted version.

## Funding

This research was supported by Jiangsu Provincial Basic Research Program Natural Science Foundation to MW (BK20200613). DL was funded by the National Social Science Fund of China (21BC042).

## Conflict of interest

The authors declare that the research was conducted in the absence of any commercial or financial relationships that could be construed as a potential conflict of interest.

## Publisher's note

All claims expressed in this article are solely those of the authors and do not necessarily represent those of their affiliated organizations, or those of the publisher, the editors and the reviewers. Any product that may be evaluated in this article, or claim that may be made by its manufacturer, is not guaranteed or endorsed by the publisher.
